# Prevalence and Molecular Typing of Carbapenemase-Producing Enterobacterales among Newborn Patients in Italy

**DOI:** 10.3390/antibiotics11040431

**Published:** 2022-03-23

**Authors:** Marilena Agosta, Daniela Bencardino, Marta Argentieri, Laura Pansani, Annamaria Sisto, Marta Luisa Ciofi Degli Atti, Carmen D’Amore, Lorenza Putignani, Pietro Bagolan, Barbara Daniela Iacobelli, Andrea Dotta, Ludovica Martini, Luca Di Chiara, Mauro Magnani, Carlo Federico Perno, Francesca Andreoni, Paola Bernaschi

**Affiliations:** 1 Microbiology and Diagnostic Immunology Unit, Department of Diagnostic and Laboratory Medicine, Bambino Gesù Children’s Hospital, IRCCS, 00165 Rome, Italy; marilena.agosta@opbg.net (M.A.); marta.argentieri@opbg.net (M.A.); laura.pansani@opbg.net (L.P.); annamaria.sisto@opbg.net (A.S.); carlofederico.perno@opbg.net (C.F.P.); 2 Department of Biomolecular Sciences, University of Urbino “Carlo Bo”, 61032 Fano, Italy; daniela.bencardino@uniurb.it (D.B.); mauro.magnani@uniurb.it (M.M.); francesca.andreoni@uniurb.it (F.A.); 3 Clinical Pathways and Epidemiology Unit, Bambino Gesù Children’s Hospital, IRCCS, 00165 Rome, Italy; marta.ciofidegliatti@opbg.net (M.L.C.D.A.); carmen.damore@opbg.net (C.D.); 4 Human Microbiome Unit, Department of Diagnostics and Laboratory Medicine, Bambino Gesù Children’s Hospital, IRCCS, 00165 Rome, Italy; lorenza.putignani@opbg.net; 5 Neonatal Surgery Unit, Medical and Surgical Department of the Fetus-Newborn-Infant, Bambino Gesù Children’s Hospital, IRCCS, 00165 Rome, Italy; pietro.bagolan@opbg.net (P.B.); bdaniela.iacobelli@opbg.net (B.D.I.); 6 Neonatal Intensive Care Unit, Medical and Surgical Department of the Fetus-Newborn-Infant, Bambino Gesù Children’s Hospital, IRCCS, 00165 Rome, Italy; andrea.dotta@opbg.net (A.D.); ludovica.martini@opbg.net (L.M.); 7 Pediatric Cardiac Intensive Care Unit, Department of Cardiology and Cardiac Surgery, Bambino Gesù Children’s Hospital, IRCCS, 00165 Rome, Italy; luca.dichiara@opbg.net

**Keywords:** Enterobacterales, carbapenem resistance, neonates, plasmid-typing, sequence type

## Abstract

The spread of carbapenemase-producing Enterobacterales (CPE), especially *Klebsiella pneumoniae* (*K. pneumoniae*) and *Escherichia coli* (*E. coli*), is a serious public health threat in pediatric hospitals. The associated risk in newborns is due to their underdeveloped immune system and limited treatment options. The aim was to estimate the prevalence and circulation of CPE among the neonatal intensive units of a major pediatric hospital in Italy and to investigate their molecular features. A total of 124 CPE were isolated from rectal swabs of 99 newborn patients at Bambino Gesù Children’s Hospital between July 2016 and December 2019. All strains were characterized by antimicrobial susceptibility testing, detection of resistance genes, and PCR-based replicon typing (PBRT). One strain for each PBRT profile of *K. pneumoniae* or *E. coli* was characterized by multilocus-sequence typing (MLST). Interestingly, the majority of strains were multidrug-resistant and carried the *bla*_NDM_ gene. A large part was characterized by a multireplicon status, and FII, A/C, FIA (15%) was the predominant. Despite the limited size of collection, MLST analysis revealed a high number of Sequence Types (STs): 14 STs among 28 *K. pneumoniae* and 8 STs among 11 *E. coli*, with the prevalence of the well-known clones ST307 and ST131, respectively. This issue indicated that some strains shared the same circulating clone. We identified a novel, so far never described, ST named ST10555, found in one *E. coli* strain. Our investigation showed a high heterogeneity of CPE circulating among neonatal units, confirming the need to monitor their dissemination in the hospital also through molecular methods.

## 1. Introduction

The spread of carbapenem-resistant Enterobacterales (CRE) is an emerging concern worldwide and, noteworthy, carbapenem-resistant gram-negative bacterial infections are counted in the WHO priority pathogen list [1,2]. Due to continuous evolution of molecular mechanisms of resistance, the epidemiology of CRE is changing, and an increasing number of people are being colonized and infected by these organisms worldwide [3,4]. These infections are associated with high mortality ranging from 40% to 50%, and their prevalence continue to increase [4]. Indeed, carbapenemase-producing Enterobacterales (CPE) typically carry genes that confer resistance to other antibiotics and they are often extensively drug-resistant or even pan-drug resistant, limiting treatment options and leading to a high rate of therapeutic failures and subsequently to fatalities [5,6]. Globally, *Klebsiella pneumoniae* carbapenemases (KPCs) are the most common transmissible genes circulating in Enterobacterales. They have the ability to hydrolyze all β-lactams, and strains carrying *bla*_KPC_ often acquire resistance to several antibiotic agents, resulting in Multidrug-Resistant Organisms (MDROs) [7]. The rapid spread of KPC-Enterobacterales is due to the successful ST258 lineage, multidrug-resistant strains of *K. pneumoniae* endemic in several countries and responsible for many outbreaks [8].

However, in the last decade, the emergence of the most recently described carbapenemase New Delhi metallo-lactamase (NDM-1) was widely described as a matter of concern in clinical settings. This carbapenemase belongs to the metallo-lactamases class (MBLs) hydrolyzing a broad range of β-lactams with the exception of monobactams such as aztreonam. Infections caused by NDM producers include urinary tract infections, peritonitis, septicemia, pulmonary infections, soft tissue infections, and device-associated infections [9]. In contrast with KPC genes, the spread of NDM-type MBLs seems to be not associated with clonal lineages, but it is mediated by different plasmid incompatibility (Inc) groups [4,9]. Indeed, the clonal spread of these microorganisms among different patients develops very easily, and resistance genes to carbapenems can be transmitted between microorganisms of different species through plasmids [10].

In this scenario, the spread of (MDROs) is particularly worrisome in the Neonatal Intensive Care Unit (NICU): in this complex care setting, the emergence and dissemination of CRE and CPE, especially *K. pneumoniae* and *E. coli*, can occur with particular frequency and serious risks for newborns [11,12,13]. The epidemiological context and, in particular, the level of endemicity in communities and care settings, play a very important role in the risk of importing isolates and/or genetic resistance determinants into a NICU. Community-acquired (CA) pathogens are introduced in the hospital through the patients (including neonates and infants) themselves, their parents, and healthcare professionals. Furthermore, in the reference NICU of a specific territorial area, the epidemiology of microorganisms is strongly influenced by patient movements from and to other NICUs. Alongside the condition for which they are transferred (e.g., malformations, surgical emergencies, etc.), they present additional risk factors for the carriage of MDRO, i.e., previous exposure to another high-risk care setting [14,15,16,17].

On the other hand, prolonged hospitalization in such fragile patients is known to predispose to hospital-acquired colonization (HAC) [18,19]. For these reasons, to monitor the spread of carbapenemase-producing organisms (CPO), different international institutions have developed numerous guidelines [20,21,22], which have also been adopted in Italy.

Current prevention strategies for carriage and infections by CPE include active surveillance using culture and/or molecular methods, adoption of contact precautions, isolation or cohorting and, in selected cases, decolonization. Several studies emphasize the importance of identifying individuals carrying antimicrobial-resistant bacteria in both the patient and healthy population [23,24]. In fact, asymptomatic rectal carriers are considered the main source of spread of multidrug-resistant organisms, especially among fragile individuals, such as pediatric ones, where colonization is a precursor to infections such as bacteraemia, pneumonia, central nervous system (CNS), and urinary tract infections [25,26]. A large amount of information is reported in the literature for adults but, to date, little is known about the epidemiology and screening strategy of CPE in children, and even less in newborns.

The aim of this study was to estimate the distribution of CPE strains isolated during a three-year period from newborn patients admitted to the NICU and cardiac intensive care unit (CICU) of a large, tertiary care pediatric Italian hospital. All CPE strains were characterized in terms of their antimicrobial resistance patterns and molecular features such as determinants and plasmid replicons. Each strain of *K. pneumoniae* and *E. coli*, representative for each PBRT profile, was further characterized by multilocus-sequence typing (MLST).

## 2. Results

### 2.1. Epidemiological Features

Between July 2016 and December 2019, 124 CPE strains were consecutively collected from 99 newborn patients (44 females and 55 males) aged from six days to one year. A large part of newborns (72%) was hospitalized in the neonatal surgical unit (NSU), 15% in neonatal intensive therapy (NIT), 6% in the sub-intensive neonatal unit (SNU), and the remaining 5% in the cardiac intensive care unit (CICU). All strains were collected from rectal swabs: 105 (84%) were isolated from Italian patients, whereas 19 (15.3%) from 14 foreign patients: three from Burundi, two from Libya, Russia, and Romania, and one from Iraq, Georgia, Central African Republic, Sierra Leone, and Ukraine. From all patients, at least one strain was isolated (one strain from each swab), with the exception of 25 children subjected to a second swab during the hospitalization stay or after the second admission. In detail, 50 strains were isolated from 25 patients, and the remaining 74 strains were isolated from 74 patients. Moreover, the major part of the strains (*n* = 103; 82%) were isolated from patients colonized during their hospitalization, whereas 21 strains (17%) were community-acquired because they were isolated from patients who were colonized at the time of their admission to the hospital.

### 2.2. Isolate Identification

Of 124 CPE strains, *K. pneumoniae* (*n* = 55; 44%), *E. coli* (*n* = 44; 35%), *Klebsiella oxytoca* (*n* = 7; 6%), *Enterobacter cloacae* (*n* = 7; 6%), *Citrobacter freundii* (*n* = 6; 5%), *Klebsiella aerogenes* (*n* = 2; 2%), *Citrobacter koseri* (*n* = 1; 1%), *Serratia marcescens* (*n* = 1; 1%), and *Morganella morganii* (*n* = 1; 1%) were identified. The most prevalent species of *K. pneumoniae* and *E. coli* were widely detected both in NICU and CICU wards, with a significant presence in NSU (*n* = 41/55 for *K. pneumoniae*; *n* = 35/44 for *E. coli*). One foreign patient carried *Citrobacter koseri* and another carried *Morganella morganii*.

### 2.3. Antimicrobial Resistance Characterization

All CPE strains were found to be resistant to nearly all antibiotics tested in this study, including imipenem (*n* = 121; 97%) and meropenem (*n* = 119; 96%). High resistance was detected towards amoxicillin–clavulanic acid (100%), cefotaxime (100%), ceftazidime (100%), and piperacillin–tazobactam (100%). The lowest rate of resistance was recorded for tigecycline (*n* = 4; 3%). All strains of *K. pneumoniae* were resistant to all the antibiotics tested, whereas those of *E. coli* were susceptible only to tigecycline. Among 21 strains related to community-acquired colonization, 12 different antibiotic patterns were recorded, showing very high variability.

All 124 CPE strains were positive for carbapenemase genes, with the prevalence of 73% for the New-Delhi Metallo-β-lactamase gene (*bla*_NDM_). In detail, the *bla*_NDM_ gene was detected in combination with *bla*_KPC_ and *bla*_VIM_ in 24 and 2 strains, respectively. By contrast, the remaining *bla*_KPC_ (*n* = 15), *bla*_VIM_ (*n* = 13), and *bla*_OXA_-48 (*n* = 3) genes were individually detected.

### 2.4. Replicon Typing

**Figure 1:**
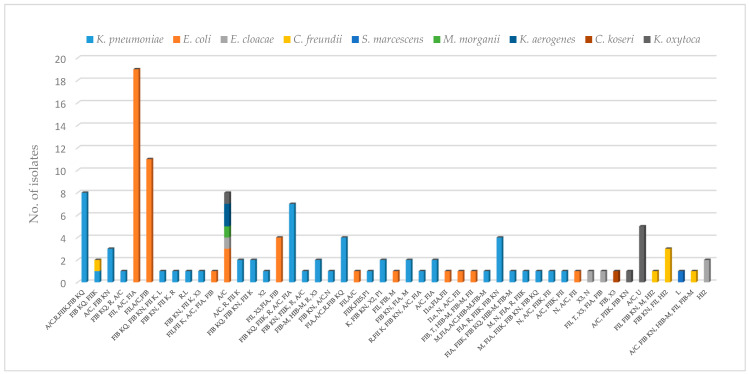
Distribution of PBRT patterns among CPE strains. Forty-nine PBRT profiles were distributed among 122 out of 124 CPE strains. All of these PBRT patterns were associated with a single species with the exception of replicon A/C (*E. coli, E. cloacae, M. morganii, K. aerogenes*, and *K. oxytoca*) and IncFIB KQ, IncFIIK (*K. pneumoniae* and *C. freundii*).

A summary of replicons detected among the 122 CPE strains is given in Figure 1. Overall, only two strains of *E. cloacae* were not typeable by the PBRT, and IncA/C was the most common Inc group (65%), followed by IncFIA, IncFII, and IncFIIK found in 43%, 39%, and 32% of isolates, respectively. In this study, 18 out of 30 replicons were identified, whereas IncHI1, IncI2, IncB0, IncP1, IncW, IncI1γ, IncFIIS, IncN2, IncX1, IncX2, IncK, and IncX4 were not detected.

The majority of isolates (*n* = 94/122; 77%) were characterized by the multireplicon status carrying three or more different Inc groups. In detail, the predominant multireplicon profiles were FII, A/C, FIA (*n* = 19; 15%) and FII, A/C, FIB (*n* = 11; 9%), both found only among *E. coli* strains. Instead, the two multireplicon profiles consisting of A/C, R, FIIK, FIB KQ and FIB KQ, FIIK, R, A/C, and FIA were the most common in *K. pneumoniae*.

In our study, we observed that all forty-nine PBRT patterns were species-specific with few exceptions. In fact, the profile composed by the single replicon A/C was found in *E. coli*, *E. cloacae*, *M. morganii*, *K. aerogenes*, and *K. oxytoca,* whereas the pattern IncFIB KQ, IncFIIK was found in *K. pneumoniae* and *C. freundii*.

### 2.5. Correlation between PBRT and Antibiotic Resistance Patterns

The distribution of PBRT and antibiotic resistance within the collected strains is reported in Table 1. Although we detected a consistent heterogeneity, 12 (24%) PBRT profiles seemed to be related with specific antibiotic-resistance patterns and the associated carbapenemase-resistance genes. For instance, the PBRT profile IncFII, IncA/C, IncFIA (indicated with the number 5) was present in 18 *E. coli* strains, and all of them reported the same antibiotic resistance pattern (AMK-AMC-FEP-CTX-CAZ-CIP-GEN-IPM-TZP-MEM) and the same carbapenemase determinant (*bla*_NDM_). This type of analysis was performed for all PBRT profiles, which included at least two strains, and numbered as 2, 13, 14, 16, 17, 19, 24, 28, 33, 46, and 49 (Table 1). Moreover, within the PBRT profiles IncFII, IncA/C, IncFIA and IncFIB KQ, IncFIIK, IncR, IncA/C, and IncFIA (numbered 5 and 17, respectively) we observed the highest number of strains that not only shared the same antibiotic-resistance pattern and related determinants, but also the same species (*E. coli* and *K. pneumoniae*, respectively), origin of patients (Italy), and type of colonization. By contrast, the highest variability was found for the PBRT profile IncA/C (number 12) wherein, out of eight strains, five different species (*E. coli, K. aerogenes, K. oxytoca, M. morganii*, and *E. cloacae*), five different antibiotic-resistance patterns, and three different carbapenemase determinant patterns were recorded.

Overall, correlations between replicons and multiple resistance profiles found in this study confirmed that plasmids are an important reservoir for the spread of resistance in Enterobacterales.

### 2.6. Multilocus-Sequence Typing

MLST analysis performed on 39 strains, each representative of a PBRT profile found in *E. coli* (*n* = 11) or *K. pneumoniae* (*n* = 28), revealed 22 STs. As described in Table 1, 14 different STs in 28 carbapenemase-producing *K. pneumoniae* isolates were found, with the prevalence of ST307 (*n* = 5; 18%), followed by ST17 (*n* = 4; 14%) and ST395 (*n*= 4; 14%). Among four *K. pneumoniae* strains isolated from foreign-born neonates, four different STs were detected (ST307, ST11, ST323, and ST1412). All strains were isolated from patients colonized upon their admission (CAC), with the exception of the strain belonging to ST323 related to hospital-acquired colonization. Moreover, these patients were hospitalized in NIT and NSU wards. Despite the modest size of collection, these findings suggest a certain degree of heterogenicity, due to the high number of circulating clones independently by origin of patient and hospitalization ward.

**Figure 2:**
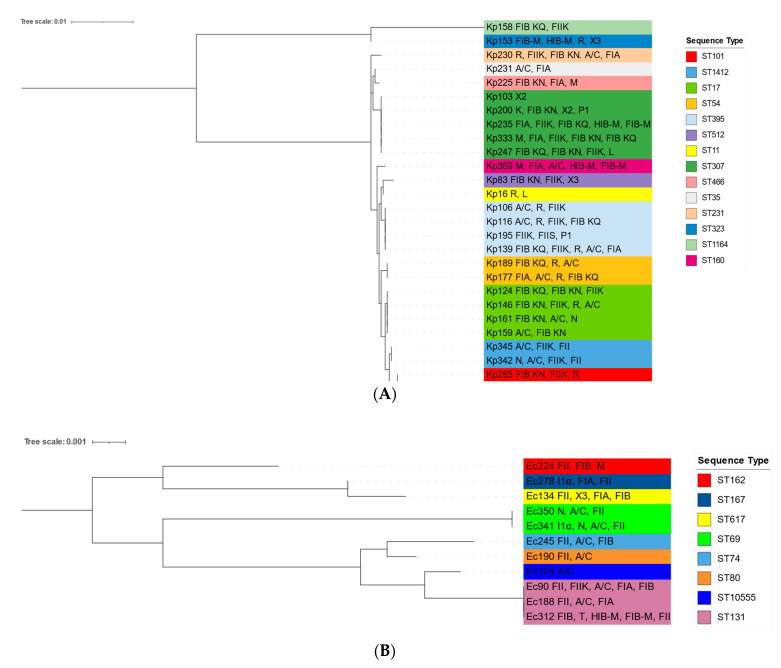
MLST-based phylogenetic trees of *K. pneumoniae* and *E. coli* strains. Trees of concatenated nucleotide sequences of seven housekeeping genes of *K. pneumoniae* (**A**) and *E. coli* (**B**) were obtained using the Maximum Likelihood method and the General Time Reversible model by MEGA software (version 10.0) with bootstrap percentages retrieved in 1000 replications. Graphical representation of both were obtained using Interactive Tree of Lifes (iTOL v.6) software. Scale bars indicate the number of nucleotide substitutions per site. For each strain, the name was reported as Kp for *K. pneumoniae* and Ec for *E. coli*. STs and replicon profiles were also indicated for each strain.

In light of this, we constructed phylogenetic trees in order to investigate the relationship among all STs identified in our analysis, and clonal relatedness of strains is shown in Figure 2. This analysis involved 28 nucleotide sequences for *K. pneumoniae* with 3018 positions in the final dataset.

Only the two strains belonging to ST323 and ST1164 seemed to be less related with the others, whereas strains typed with the most common identified ST307 showed higher phylogenetic relation with strains belonging to ST35 and ST466. Thus, all *K. pneumoniae* strains of our collection showed a clonal relatedness with the exception of two strains that were not genetically related with the main cluster. All strains grouped in this main cluster are well-known to be responsible for carbapenem resistance spread.

On the other hand, eight different STs were observed among 11 carbapenemase-producing *E. coli*, and ST131 was the main, found in three strains (two isolated from foreign patients). Instead, two strains belonged to ST69, and the remaining STs (ST617, ST80, ST74, ST162, ST167, and the novel ST10555) were individually found. Moreover, all foreign patients were hospitalized in the NSU ward, and four out of five strains isolated from them belonged to different STs (ST131, ST617, ST167, and ST69), reporting community-acquired colonization. By contrast, all *E. coli* strains isolated from Italian patients were associated with hospital-acquired colonization concerning not only the NSU ward but also the other wards.

Notwithstanding the low number of strains typed by MLST, we detected a wide diversity of STs in our *E. coli* collection as confirmed by the phylogenetic analysis. It involved 11 nucleotide sequences with a total of 3423 positions in the final dataset and revealed a higher relation among strains belonging to ST131, the novel ST10555, ST80, and ST74 (Figure 2). Of note, a main cluster was not identified, but we observed an interesting similarity of the novel ST10555 with the most common ST131. The latter is known to be an emerging carbapenemase-producing clone, suggesting the ability of the novel ST10555 to disseminate carbapenemase genes.

### 2.7. The Novel ST10555

Interestingly, a new sequence type was identified among *E. coli* clinical isolates (named *E. coli*_104) as shown in Table 1 and Figure 2. This strain was isolated from an Italian male patient (five months of age) in 2017. The isolate exhibited the A/C replicon and the following antibiotic-resistance profile AMK-AMC-FEP-CTX-CAZ-CIP-GEN-IPM-TZP-SXT-MEM; carbapenem resistance was confirmed by the detection of *bla*_NDM_ gene. It revealed a novel allelic variant for the *recA* gene (recorded as 772) confirmed through whole-genome sequencing. Successively, the entire genome was submitted to EnteroBase database at https://enterobase.warwick.ac.uk/species/index/ecoli (accessed on 21 March 2022), resulting in the associated ST10555 (*adk* 13, *fumC* 52, *gyrB* 10, *icd* 14, *mdh* 17, *purA* 25, *recA* 772). Moreover, we used genomic analysis tools to explore the whole-genome sequencing data. Through ResFinder 4.1 we confirmed the resistance observed phenotypically and found additional resistance towards tobramycin, cefoxitin, ampicillin, and ertapenem. The same analysis revealed the following resistance genes: *aac(6′)-Ib3* (amikacin, tobramycin), *aph(3′)-VI* (amikacin), *rmtC* (amikacin, gentamicin, tobramycin, kanamycin), *bla*_CMY-6_ (amoxicillin, amoxicillin–clavulanic acid, ampicillin, cefotaxime, ceftazidime, piperacillin–tazobactam), *bla*_NDM-1_ (amoxicillin, amoxicillin + clavulanic acid, ampicillin, cefepime, cefotaxime, cefoxitin, ceftazidime, ertapenem, imipenem, meropenem), and *bla*_CTX-M-15_ (amoxicillin, ampicillin, cefepime, cefotaxime, ceftazidime, piperacillin). Moreover, through PlasmidFinder 2.0 and pMLST-2.0 Server, we confirmed that this isolate belonged to IncC group and to the profile IncA/C PMLST, respectively.

Finally, SerotypeFinder 2.0 Server revealed the serotype H6 encoded by the *flic* gene (99.94% of identity) as well as the serotype O2 encoded by *wzy* and *wzx* genes, with the identities of 98.03% and 98.65%, respectively. The genetic features of this newly emerged strain type highlight the risk associated with its circulation, especially in terms of antibiotic resistance spread that is a matter of concern in the neonatal population.

## 3. Discussion

Although CPE infections are endemic in Italy and neonates and infants are considered patients at high risk, few studies have been carried out to evaluate their dissemination among neonatal intensive units [12,13,27,28,29]. Retrospective studies carried out among the NICUs of different pediatric Italian hospitals reported high rates of colonization by strains with important molecular features, highlighting the role of risk factors on infection incidence. For instance, Montagnani and colleagues confirmed the immunosuppressive state of children with hematologic/oncologic conditions, invasive devices, history of surgery or hospitalization, and prior use of antibiotics as important risk factors associated with CPE infections [27]. Instead, Mammina and colleagues showed that feeding by formula was significantly associated with colonization due to cross-contamination and poor infection control procedures [30]. On the other hand, a more recent study demonstrated that the efficacy of screening programs associated with proactive measures to control cross-contamination and hospital-acquired colonization are effective to reduce the spread of CPE also in the pediatric setting [13]. Moreover, these studies were useful also to collect information about the emerging clones circulating among neonatal wards, tracing the evolution of their molecular aspects.

Here, we described CPE distribution in neonatal wards, including NICU, and a CICU of a major tertiary pediatric referral hospital in Italy, where foreign patients are also frequently admitted. The first interesting point was the high variability of CPE detected in terms of isolates and associated molecular features. In accordance with other studies, within the variety of species detected in our collection and widely associated with neonatal colonization, *K. pneumoniae* and *E. coli* were the most common [12,13,31,32,33]. Most strains (76%) exhibited resistance to at least one agent of three or more antimicrobial categories tested in our panel. Hence, they were classified as multidrug-resistant (MDR) as described by Majorakos et al. [34], increasing the risk of treatment failure. To note, almost all strains were susceptible to tigecycline (97%). This was in agreement with results reported by the SENTRY surveillance program where a good activity of tigecycline against these microorganisms was indicated [35]. Conversely, other studies reported higher levels of resistance towards this antimicrobial agent [27], and recent investigations suggest an improved efficacy of tigecycline against carbapenem-resistant Enterobacterales in combination with colistin [36] or with ceftazidime–avibactam [37]. These findings suggest the need to monitor the use of this or other last-resort antibiotics in order to limit the impairment of treatment due to increased levels of resistance.

Moreover, CPE strains have the potential to spread widely due to the localization of carbapenemase genes on mobile genetic elements, which further complicate the treatment, increasing mortality and morbidity rates [38,39]. Overall, 26% of strains coharbored a combination of two resistance genes, thus contributing to increased risk in terms of public health. It was not surprising that the predominant determinant of carbapenem resistance found in our study was *bla*_NDM_. Indeed, the rapid dissemination of New Delhi metallo-beta-lactamase (NDM)-producing carbapenem-resistant Enterobacterales have been largely detected in central Italy in the last years [40]. This spread of NDM-producing strains confirms a significant change in CPE epidemiology consisting in replacement of previous endemicity of *bla*_KPC_ genes.

It is well-known that *K. pneumoniae* and *E. coli* are the main drivers of this rapid and consistent spread, and the dissemination of NDM is generally mediated by plasmids with a variety of replicon types [41]. In accordance with the literature, the predominant IncA/C plasmid (66%) detected in the present study was strongly associated with *bla*_NDM_ genes [42,43]. This plasmid type, identified in all enterobacterial species, possesses a broad host range of replication and has been identified not only from human but also from animal isolates [44,45]. For all of these reasons, the IncA/C-type plasmid is considered an increasing threat to public health. Moreover, the majority of isolates (77%) carried three or more replicons defining the multireplicon-status, with a high percentage of the IncF family that it is known to be restricted to Enterobacterales. The multireplicon status promotes acquisition of plasmids carrying incompatible replicons, enlarging the host range replication also between different species [46]. A typical multireplicon IncF plasmid carries FII, FIA, and FIB, as also observed in our collection, confirming their large diffusion among clinical Enterobacterales [46,47]. From a functional point of view, FII is silent, whereas the activity of FIA as well as that of FIB is exclusively related to enteric bacteria [46].

Additional interesting information about the diversity of strains investigated in the present study was revealed by multilocus-sequence typing analysis. The identification of ST was performed for a single strain representative for each PBRT pattern in order to obtain preliminary information about the clones circulating in the neonatal intensive unit. Of all the obtained STs, ST307 was the most common in *K. pneumoniae*, confirming the recent and rapid spread of this emerging clone in Italy [48,49,50]. Noteworthy, the diffusion of ST307 in Italy traces the evolution of the current epidemiological change because it seems to replace the global ST258 and its endemic Italian variant ST512. Indeed, after the first outbreak occurred in Sicily (Italy) in 2008 [51], where KPC-3-producing *K. pneumoniae* ST258 was mainly responsible, a second surveillance carried out in 2014 revealed the emerging of KPC-3-producing strains ST307 [29,52]. As also detected in our investigation, ST307 is a novel distinctive lineage carrying *bla*_KPC_ determinants [49], even if in our study we found it in association with *bla*_NDM_ or *bla*_OXA-48_, suggesting the potential to acquire advantageous features for the adaption to clinical niches. Furthermore, the increased detection of ST307 also in other countries in the last years suggests not only its pivotal role in the spread of antibiotic resistance but also the potential to become one of the most clinically relevant clones. Our data confirmed the increasing CPE prevalence in neonates and infants and showed that the molecular characteristics of strains are evolving. Moreover, CPE colonization represents an important reservoir for nosocomial infections due to their stronger virulence and transmission. Hence, an active screening also through molecular typing is very useful to classify genotypes and is a priority in vulnerable patients like newborns, where multiple-antibiotic resistance can result in failed infection treatment.

On the other hand, *E. coli* strains characterized by MLST revealed several STs, but this collection was too limited, and the frequency of these clones could be underestimated. Among them, ST131, known to be responsible for hospital- and community-acquired urinary tract infections as well as bloodstream infections, was most commonly detected. Most strains belonging to ST131 are MDR, therefore limiting therapeutic options that cause recurrent infections [53].

The identification of the novel ST10555 highlights the variability observed among *E. coli* strains despite the low size of collection. It was isolated from an Italian patient and exhibited resistance towards all of antibiotics tested in our study, with the exception for tigecycline. Whole-genome analysis revealed the carriage of other determinants besides *bla*_NDM_, confirming the risk associated with its distribution among newborn patients. This novel ST was phylogenetically related with ST131, described as a high-risk clone due to its role in antibiotic resistance spread, confirming the continuous emergence of new clones with potential to colonize clinical niches (Figure 2).

Notwithstanding the limited size of our collection, the richness and variability of genetic repertoire showed by these strains made more evident the dynamic evolution of CPE and, consequently, the importance of monitoring their distribution in healthcare settings.

From a health perspective, the results of this study highlight the urgency of addressing the surveillance of CPE distribution in pediatric hospitals. One important challenge for hospitals consists of collaborative efforts with different and external professional figures of health sciences. Indeed, in addition to the commonly reported guidelines implemented by WHO protocols to manage the prescription of antibiotics in therapeutic plans, strict cooperation among diagnostic laboratories, hospitals, and research institutes is strongly recommended to reduce the spread of these dangerous strains and prevent the emergence of serious infections in newborns. Considering this, we highlight the usefulness of molecular typing to better understand the distribution of resistant strains in clinical settings, especially where patients at risk, like neonates and children, are hospitalized.

## 4. Materials and Methods

### 4.1. Ethics Statement

The Institutional Review Board (IRB) of the IRCCS Bambino Gesù Children’s Hospital approved the study protocol (n.2156_OPBG_2020). As the data in this study were collected and analyzed retrospectively, the study did not infringe upon the rights or welfare of the patients and did not require their consent.

### 4.2. Study Design

This study was a retrospective investigation carried out from July 2016 to December 2019. A total of 9914 surveillance rectal swabs were performed for CPE screening from newborn patients hospitalized or admitted to the neonatal surgical unit (NSU), neonatal intensive therapy (NIT), the sub-intensive neonatal unit (SNU), and the cardiac intensive care unit (CICU) of Bambino Gesù Children’s Hospital in Rome (Italy), according to the active surveillance protocol issued by the hospital infection control committee. Wards considered in this study were those in which a possible transfer of patients frequently occurred during their hospital stay.

Strains were considered community-acquired when isolated from patients that were colonized at the time of their admission to the hospital. Otherwise, strains isolated from hospitalized patients were classified as hospital-acquired.

We collected demographic and clinical information about the enrolled patients from electronic medical records, including sex, age, geographical origin, the date of rectal swab collection, and department hospitalization (e.g., neonatal surgical unit, neonatal intensive therapy, sub-intensive neonatal unit, and cardiac intensive care unit). After isolation and identification, all CPE strains were characterized by antimicrobial susceptibility testing, detection of carbapenemase-encoding genes, and PBRT. Finally, one single strain of *K. pneumoniae* or *E. coli* representative for each PBRT profile was analyzed by MLST.

### 4.3. Microbiological Cultures and Antibiotic Susceptibility Testing

For cultural screening, each swab was inoculated on a set of two plates: MacConkey agar plate (bioMérieux, Craponne, France) with a 10 μg meropenem disk (Oxoid, Basingstoke, UK), according to EUCAST guidelines for the detection of resistance mechanisms (https://www.eucast.org/resistance_mechanisms/ (accessed on 21 February 2022)) and a CHROMID^®^ CARBA plate (bioMérieux, Craponne, France), implemented in our routine laboratory practice.

Plates were incubated at 37 °C overnight. All the morphologically different colonies growing into the meropenem disk halo (zone diameter < 28 mm) and on the selective chromogenic medium were picked up and subcultured for purity onto a MacConkey agar plate (bioMérieux, Craponne, France) [54,55].

Isolated colonies were identified by using Matrix-Assisted Laser Desorption Ionization–Time-of-Flight Mass Spectrometry (MALDI-TOF, Bruker Daltonics, Bremen, Germany). Antimicrobial susceptibility testing was performed using the automated Vitek 2 (bioMérieux, Craponne, France) instrument. The following antimicrobial agents were tested with the automated system: amikacin (AMK), amoxicillin–clavulanic acid (AMC), cefepime (FEP), cefotaxime (CTX), ceftazidime (CAZ), ciprofloxacin (CIP), gentamicin (GEN), imipenem (IPM), meropenem (MEM), piperacillin–tazobactam (TZP), tigecycline (TGC), trimethoprim–sulfamethoxazole (SXT).

The MIC value of meropenem was confirmed with gradient test methods by MIC Test Strip (Liofilchem, Roseto degli Abruzzi, Italy) on Muller–Hinton agar (bioMérieux, Craponne, France), incubated at 37 °C overnight. The isolates were identified as resistant to carbapenems according to clinical breakpoints based on the European Committee on Antimicrobial Susceptibility Testing (EUCAST) breakpoints tables. We adopted the updated EUCAST breakpoints tables (version 6.0 to version 9.0, from 2016 to 2019) (https://www.eucast.org/clinical_breakpoints/ (accessed on 21 February 2022)).

### 4.4. PCR-Based Methods for Carbapenemase Genes

The Xpert Carba-R molecular assay (Cepheid, Sunnyvale, CA, USA) was performed to confirm the presence of resistance genes to carbapenems. Briefly, subcultured isolated colonies were diluted in 0.45% saline to the turbidity of a 0.5 McFarland standard. Ten microliters of the suspension were inoculated into a 5 mL sample reagent vial and vortexed for 30 s. Finally, 1.7 mL of this suspension was transferred into an Xpert Carba-R cartridge using a disposable transfer pipette. The cartridge was loaded onto the GeneXpert system, and the assay was performed. The test, based on automated real-time PCR, is designed for rapid detection and differentiation of the *bla*_KPC_, *bla*_NDM_, *bla*_VIM_, *bla*_OXA-48_, and *bla*_IMP-1_ gene sequences associated with carbapenem-non-susceptible Gram-negative bacteria. The results were interpreted by the GeneXpert System from measured fluorescent signals.

### 4.5. Bacterial DNA Extraction and Plasmid Typing

Total DNA of isolated colonies that were positive for carbapenemase genes was extracted using an EZ1 DNA Tissue Kit (Qiagen, Hilden, Germany). Briefly, isolated colonies were diluted in 0.45% saline to the turbidity of 0.5 McFarland standard, and 200 µL of the suspension was transferred into a 2 mL vial. The vial and prefilled reagent cartridges were loaded onto the EZ1 Advanced XL (Qiagen, Hilden, Germany) instrument, and the protocol for automated purification of bacterial DNA, using magnetic particle technology, was started. DNA was eluted in a final volume of 100 µL and stored at −20 °C until use.

One µL of the extracted DNA was used for plasmid typing using a PCR-based replicon typing (PBRT) kit 2.0 (Diatheva, Fano, Italy). This novel PBRT assay, consisting of eight multiplex PCRs, is able to detect 30 different replicons of the main plasmid families in Enterobacterales [56]. This kit was used following the manufacturer’s instructions, including positive controls. Amplification products were resolved and visualized directly on a closed ready-to-use 2.2% agarose gel-cassette system (FlashGel-Lonza, Basel, Switzerland) using the 100 bp FlashGel DNA marker. The obtained fragments were compared to positive controls of each multiplex PCRs.

### 4.6. Multilocus Sequence Typing

Multilocus sequence typing (MLST) was performed to subtype *K. pneumoniae* and *E. coli* strains using housekeeping genes. For the former, the seven gene fragments (*gapA, infB, mdh, pgi, phoE, rpoB,* and *tonB*) were amplified by PCR and sequenced as described by protocol 2 of the Institute Pasteur Klebsiella MLST database (https://bigsdb.pasteur.fr/klebsiella/primers_used.html (accessed on 21 February 2022)). Instead, the seven housekeeping genes (*adk, fumC, gyrB, icd, mdh, purA,* and *recA*) to type *E. coli* strains were selected from the Enterobase MLST database (http://enterobase.warwick.ac.uk/species/index/ecoli (accessed on 28 February 2022). Primer sequences and reaction conditions were previously described by Wirth and colleagues (2006) [57].

All amplicons were sequenced using a BigDye Terminator v. 1.1 Cycle Sequencing kit on an ABI PRISM^®^ 310 Genetic Analyzer (Thermo Fisher Scientific, Waltham, MA, USA). The alignment between sequences and the related reference was carried out using Unipro UGene version 38.0 software [58]. The allele numbers and the sequence types (STs) were determined through the corresponding MLST database. Finally, phylogenetic analyses of concatenated allelic variants were performed using Molecular Evolutionary Genetics Analysis (MEGA) software version 10.0. The evolutionary history was inferred using the Maximum Likelihood method and the General Time Reversible model. Initial trees for the heuristic search were obtained applying the Neighbor-Joining method to a matrix of pairwise distances estimated using the Maximum Composite Likelihood (MCL) approach. Branch quality was evaluated using a bootstrap test with 1000 replicates [59]. Graphical representation of both were obtained using Interactive Tree Of Lifes (iTOL v.6) software [60].

### 4.7. Whole-Genome Sequencing (WGS)

Whole-genome sequencing was performed in accordance with Lindsted et al. (2018) [61] for *E. coli*_104 strain, which revealed a new *recA* allele by MLST analysis. Briefly, genomic DNA concentration was determined using a Qubit dsDNA BR Assay Kit and a Qubit 2.0 Fluorometer (Thermo Fisher Scientific, Waltham, USA) in order to obtain a DNA input concentration between 100 and 500 ng. Library preparation was performed using an Illumina DNA Prep Library Kit (Illumina, Berlin, Germany) according to the manufacturer’s instructions. The reference strain of *E. coli* ATCC 25922 was used as sequencing quality control. Sequencing was performed using a MiSeq Reagent Micro Kit on an Illumina MiSeq desktop platform (Illumina Inc., San Diego, CA, USA) for ~20 h to produce paired-end sequences (2 × 300 base pair).

Raw Illumina reads were paired and assessed for sufficient coverage (≥Q30), and bases with low quality (<Q30) were discarded. Finally, the reads in FASTQ format were uploaded and submitted to the EnteroBase database (https://enterobase.warwick.ac.uk/species/index/ecoli (accessed on 21 February 2022)). High-throughput sequencing data were submitted to the Sequence Read Archive (SRA) (GeneBank accession number SUB10115582).

Futhermore, raw reads were analyzed using a bioinformatics tool of the Center for Genomic Epidemiology such as ResFinder 4.1 [62], PlasmidFinder 2.0 [63], pMLST-2.0 Server [63], and SerotypeFinder 2.0 Server [64].

## 5. Conclusions

Great attention should be given to high prevalence of CPE in NICUs and CICU due to the vulnerability of newborn patients. The molecular features of investigated strains showed their potential to be a serious threat to the health of neonates and infants. Our study contributed to increasing the knowledge concerning CPE circulation in Italian pediatric hospitals, and it highlighted the need to strengthen control measures to avoid their spread among wards. This is crucial to avoid complications for the treatment of neonatal infections. Moreover, introducing molecular typing methods in clinical routine procedures is becoming more and more necessary to monitor the spread of high-risk clones, to track their circulation, and to promptly identify emerging clones with advantageous ability to colonize clinical niches. We provided the evidence that PBRT can be commonly integrated within the diagnostic routine of a hospital in order to obtain important information about the epidemiology of circulating strains. Data concerning replicons harbored by MDROs in general, and CPE in our case study, represent an added value for a successful surveillance program. PBRT allows clinicians to prevent or monitor the spread of strains with potential ability to acquire and disseminate resistance genes through plasmids among wards. On the other hand, we used MLST because it enabled us to complete the description of the molecular profile of circulating clones, and the phylogenetic tree was also useful for comparing the novel identified ST with the well-known others. Moreover, typing housekeeping genes can be a very useful way to identify the dominance of a specific lineage responsible for an important clinical outbreak.

## Figures and Tables

**Table 1:** Genotypic and phenotypic profile of CPE strains isolated from rectal swabs.

Strains ^a^	Age ^b^	Sex	Origin ^c^	CAC or HAC ^d^	Clinical Ward ^e^	Date	Species	Antibiotic Resistance Pattern ^f^	Genes	PBRT ^g^	ST
84-A	5 mths	M	Russia	CAC	NSU	6/9/17	*K. pneumoniae*	AMK-AMC-FEP-CTX-CAZ-CIP-GEN-IPM-TZP-SXT-MEM	*bla*_KPC_ + *bla*_NDM_	(1) A/C, R, FIIK, FIB KQ	
116	24 days	M	Italy	HAC	NIT	8/7/17					395
127	6 days	F			NSU	8/14/17					
123	14 days	M				8/14/17					
145	2 mths	M			SNU	10/2/17					
172	19 days	F				11/6/17					
122	11 days	F			NIT	8/14/17		AMK-AMC-FEP-CTX-CAZ-CIP-GEN-IPM-TZP-MEM			
126-D	1 mth	M			NSU	8/14/17		AMK-AMC-FEP-CTX-CAZ-CIP-GEN-IPM-TZP-TGC-SXT-MEM			
158	2 mths	F	Italy	CAC	NIT	10/14/17	*K. pneumoniae*	AMC-CTX-CAZ-IPM-TZP-MEM	*bla*_KPC_	(2) FIB KQ, FIIK	1164
155	4 mths			HAC	NSU	10/9/17	*C. freundii*				
159	2 mths	M	Italy	HAC	SNU	10/16/17	*K. pneumoniae*	AMK-AMC-FEP-CTX-CAZ-GEN-IPM-TZP-MEM	*bla*_NDM_	(3) A/C, FIB KN	17
168a-L	13 days				NSU						
160	20 days							AMK-AMC-FEP-CTX-CAZ-GEN-IPM-TZP-SXT-MEM			
189	3 mths	F	Italy	HAC	NIT	12/11/17	*K. pneumoniae*	AMK-AMC-FEP-CTX-CAZ-GEN-IPM-TZP-MEM	*bla*_NDM_	(4) FIB KQ, R, A/C	54
167-G	4 mths	F	Italy	HAC	NSU	10/16/17	*E. coli*	AMK-AMC-FEP-CTX-CAZ-CIP-GEN-IPM-TZP-SXT-MEM	*bla*_NDM_	(5) FII, A/C, FIA	
138-D	2 mths	M				9/18/17		AMK-AMC-FEP-CTX-CAZ-CIP-GEN-IPM-TZP-MEM			
187-O	2 mths	F				12/4/17					
188	15 days	F			SNU	12/11/17					131
191	4 mths	M			NSU	12/11/17					
204-R	1 mth	M				2/5/18					
207-Q	2 mths	M				2/9/18					
211-T	17 days	F				2/19/18					
214	18 days	F				2/26/18					
215	1 mth	M				2/26/18					
216	2 mths	M				2/26/18					
218-U	2 mths	F				2/26/18					
222	3 mths	M			NIT	3/5/18					
227	1 mth	M			SNU	3/12/18					
226-Z	2 mths	F			NSU	3/12/18					
228-S	1 year	M				3/12/18					
232	6 days	M				3/19/18					
240-Y	1 mth	M				4/16/18					
274-Z	7 mths	F				8/8/18					
163-M	1 mth	M	Italy	HAC	NSU	10/16/17	*E. coli*	AMK-AMC-FEP-CTX-CAZ-GEN-IPM-TZP-MEM	*bla*_NDM_	(6) FII, A/C, FIB	
168b-L	13 days	M			NSU	10/16/17					
169-I	1 mth	F			NSU	10/16/17					
210	1 mths	M			NIT	2/19/18					
245	6 mths	F			NIT	4/23/18					74
250	29 days	M			NSU	5/7/18					
261	2 mths	F		CAC	NIT	6/30/18		AMC-FEP-CTX-CAZ-IPM-TZP-SXT	*bla*_VIM_		
275	4 mths	M		HAC	NSU	8/20/18					
266	14 days	M			SNU	7/25/18		AMC-FEP-CTX-CAZ-IPM-TZP-SXT-MEM	*bla*_VIM_		
336	1 year	M	S. Leone		NSU	7/15/19			*bla*_NDM_		
199-P	3 mths	M	Italy		NIT	1/10/18		AMK-AMC-FEP-CTX-CAZ-TZP	*bla*_NDM_		
247	5 mths	F	Romania	CAC	NIT	4/28/18	*K. pneumoniae*	AMC-FEP-CTX-CAZ-CIP-GEN-IPM-TZP-SXT	*bla*_OXA-48_	(7) FIB KQ, FIB KN, FII K, L	307
285	1 mth	M	Italy	CAC	NIT	9/28/18	*K. pneumoniae*	AMK-AMC-FEP-CTX-CAZ-CIP-GEN-IPM-TZP-MEM	*bla*_KPC_	(8) FIB KN, FII K, R	101
16	1 mth	F	Romania	CAC	NSU	9/22/16	*K. pneumoniae*	AMK-AMC-FEP-CTX-CAZ-CIP-GEN-IPM-TZP-TGC-MEM	*bla*_OXA-48_	(9) R, L	11
83	1 mths	M	Italy	HAC	NSU	6/5/17	*K. pneumoniae*	AMK-AMC-FEP-CTX-CAZ-CIP-IPM-TZP-MEM	*bla*_KPC_	(10) FIB KN, FII K, X3	512
90-A	5 mths	M	Russia	HAC	NSU	6/19/17	*E. coli*	AMK-AMC-FEP-CTX-CAZ-CIP-GEN-IPM-TZP-SXT-MEM	*bla*_NDM_	(11) FII, FII K, A/C, FIA, FIB	131
104-B	5 mths	M	Italy	HAC	NSU	7/18/17	*E. coli*	AMK-AMC-FEP-CTX-CAZ-CIP-GEN-IPM-TZP-SXT-MEM	*bla*_NDM_	(12) A/C	10555
129	1 mth	M				8/14/17	*K. aerogenes*	AMK-AMC-FEP-CTX-CAZ-GEN-IPM-TZP-MEM			
130	15 days	M				8/18/17	*E. coli*				
131-C	1 mth	F				8/21/17					
183	6 mths	M				11/27/17	*K. aerogenes*		*bla*_KPC_+*bla*_NDM_		
229	7 mths	M				3/12/18	*K. oxytoca*	AMC-CTX-CAZ-IPM-TZP-MEM	*bla*_NDM_		
221-K	1 year	M	Russia			3/2/18	*M. morganii*	AMK-AMC-CTX-CAZ-GEN-IPM-MEM	*bla*_NDM_+*bla*_VIM_		
246	4 mths	M	Italy			4/26/18	*E. cloacae*	AMK-AMC-FEP-CTX-CAZ-GEN-TZP-MEM	*bla*_NDM_		
106	1 mth	M	Italy	HAC	NSU	7/24/17	*K. pneumoniae*	AMK-AMC-FEP-CTX-CAZ-CIP-GEN-IPM-TZP-SXT-MEM	*bla*_KPC_+*bla*_NDM_	(13) A/C, R, FII K	395
117-C	1 mth	F				8/7/17					
124	11 days	F	Italy	HAC	NSU	8/14/17	*K. pneumoniae*	AMC-CTX-CAZ-IPM-TZP-MEM	*bla*_KPC_	(14) FIB KQ, FIB KN, FII K	17
125	5 days					8/14/17					
103-B	5 mths	M	Italy	HAC	NSU	7/17/17	*K. pneumoniae*	AMC-FEP-CTX-CAZ-CIP-GEN-IPM-TZP-SXT	*bla*_KPC_	(15) X2	307
134-E	1 mth	F	Burundi	CAC	NSU	8/30/17	*E. coli*	AMC-FEP-CTX-CAZ-CIP-GEN-IPM-TZP-SXT-MEM	*bla*_NDM_	(16) FII, X3, FIA, FIB	617
137-F	1 mth					9/4/17					
166-N	1 mth		Italy	HAC		10/16/17					
174	5 mths		Libya			11/13/17					
139	3 mths	M	Italy	HAC	NSU	9/18/17	*K. pneumoniae*	AMK-AMC-FEP-CTX-CAZ-CIP-GEN-IPM-TZP-SXT-MEM	*bla*_KPC_+*bla*_NDM_	(17) FIB KQ, FIIK, R, A/C, FIA	395
144-H	4 mths	F				10/2/17					
141	13 days	F				10/2/17					
143-G	4 mths	F				10/2/17					
162-M	1 mth	M				10/16/17					
171-N	1 mth	F				10/23/17					
209-S	11mths	M				2/12/18					
146-I	16 days	F	Italy	HAC	NSU	10/2/17	*K. pneumoniae*	AMK-AMC-FEP-CTX-CAZ-GEN-IPM-TZP-MEM	*bla*_KPC_+*bla*_NDM_	(18) FIB KN, FIIK, R, A/C	17
153-F	3 mths	F	Burundi	HAC	NSU	10/9/17	*K. pneumoniae*	AMC-FEP-CTX-CAZ-CIP-IPM-TZP-SXT-MEM	*bla*_NDM_	(19) FIB-M, HIB-M, R, X3	323
154-E	3 mths					10/9/17					
161-O	17 days	F	Italy	HAC	NSU	10/16/17	*K. pneumoniae*	AMK-AMC-FEP-CTX-CAZ-GEN-IPM-TZP-TGC-MEM	*bla*_NDM_	(20) FIB KN, A/C, N	17
177-P	2 mths	M	Italy	HAC	NSU	11/20/17	*K. pneumoniae*	AMK-AMC-FEP-CTX-CAZ-GEN-IPM-TZP-MEM	*bla*_NDM_	(21) FIA, A/C, R, FIB KQ	54
178	26 days					11/20/17			*bla*_KPC_+*bla*_NDM_		
181-Q	13 days					11/27/17			*bla*_NDM_		
182	19 days					11/27/17			*bla*_NDM_		
190-H	6 mths	F	Italy	HAC	NSU	12/11/17	*E. coli*	AMK-AMC-FEP-CTX-CAZ-GEN-TZP-SXT-MEM	*bla*_NDM_	(22) FII, A/C	80
195-R	16 days	M	Italy	HAC	NSU	1/3/18	*K. pneumoniae*	AMK-AMC-FEP-CTX-CAZ-CIP-GEN-IPM-TZP-TGC-SXT-MEM	*bla*_KPC_ + *bla*_NDM_	(23) FIIK, FIIS, P1	395
200	9 mths	M	Italy	CAC	NSU	1/17/18	*K. pneumoniae*	AMC-FEP-CTX-CAZ-CIP-GEN-IPM-TZP-SXT-MEM	*bla*_KPC_	(24) K, FIB KN, X2, P1	307
289	2 mths			HAC	CICU	10/15/18					
224-V	6 mths	M	Italy	HAC	CICU	3/11/18	*E. coli*	AMC-FEP-CTX-CAZ-IPM-TZP-SXT-MEM	*bla*_VIM_	(25) FII, FIB, M	162
225-V	6 mths	M	Italy	HAC	CICU	3/11/18	*K. pneumoniae*	AMC-FEP-CTX-CAZ-IPM-TZP-SXT-MEM	*bla*_NDM_ + *bla*_VIM_	(26) FIB KN, FIA, M	466
257	20 days	F			CICU	6/13/18		AMC-FEP-CTX-CAZ-GEN-IPM-TZP-SXT-MEM	*bla*_NDM_		
230-U	3 mths	F	Italy	HAC	NSU	3/12/18	*K. pneumoniae*	AMK-AMC-FEP-CTX-CAZ-CIP-GEN-IPM-TZP-SXT-MEM	*bla*_NDM_	(27) R, FII K, FIB KN, A/C, FIA	231
231-T	1 mth	F	Italy	CAC	NSU	3/18/18	*K. pneumoniae*	AMK-AMC-FEP-CTX-CAZ-GEN-IPM-TZP-MEM	*bla*_NDM_	(28) A/C, FIA	35
237-Y	23 days	M		HAC		4/9/18					
278	2 mths	F	C.A.R.	CAC	NSU	9/11/18	*E. coli*	AMK-AMC-FEP-CTX-CAZ-CIP-GEN-IPM-TZP-SXT-MEM	*bla*_NDM_	(29) I1α, FIA, FII	167
341-X	3 mths	F	Ukraine	CAC	NSU	8/4/19	*E. coli*	AMK-AMC-FEP-CTX-CAZ-CIP-GEN-IPM-TZP-SXT-MEM	*bla*_NDM_	(30) I1α, N, A/C, FII	69
312	7 mths	F	Georgia	CAC	NSU	3/9/19	*E. coli*	AMC-FEP-CTX-CAZ-IPM-TZP-SXT-MEM	*bla*_NDM_	(31) FIB, T, HIB-M, FIB-M, FII	131
369-W	4 mths	F	Italy	HAC	NSU	10/29/19	*K. pneumoniae*	AMK-AMC-FEP-CTX-CAZ-GEN-IPM-TZP-MEM	*bla*_NDM_	(32) M, FIA, A/C, HIB-M, FIB-M	160
288	3 mths	F	Italy	CAC	NSU	10/11/18	*K. pneumoniae*	AMK-AMC-FEP-CTX-CAZ-CIP-GEN-IPM-TZP-MEM	*bla*_KPC_	(33) FIA, R, FIIK, FIB KN	101
298	2 mths	M				12/3/18					
308	6 mths	F				2/21/19					
393	3 mths	M	Iraq	HAC	CICU	1/28/20					
235	3 mths	F	Italy	CAC	NSU	4/4/18	*K. pneumoniae*	AMC-CTX-CAZ-CIP-IPM-TZP-MEM	*bla*_KPC_	(34) FIA, FIIK, FIB KQ, HIB-M, FIB-M	307
304	6 days	F	Italy	CAC	NSU	1/29/19	*K. pneumoniae*	AMK-AMC-FEP-CTX-CAZ-CIP-GEN-IPM-TZP-MEM	*bla*_KPC_	(35) M, N, FIA, R, FIIK	101
333	2 mths	F	Italy	HAC	NIT	7/1/19	*K. pneumoniae*	AMK-AMC-FEP-CTX-CAZ-CIP-GEN-IPM-TZP-SXT-MEM	*bla*_KPC_ + *bla*_NDM_	(36) M, FIA, FIIK, FIB KN, FIB KQ	307
342-X	3 mths	F	Ukraine	CAC	NSU	8/4/19	*K. pneumoniae*	AMC-FEP-CTX-CAZ-CIP-GEN-IPM-TZP-SXT-MEM	*bla*_NDM_	(37) N, A/C, FIIK, FII	1412
345	22 days	M	Italy	HAC	NSU	8/19/19	*K. pneumoniae*	AMC-FEP-CTX-CAZ-CIP-GEN-IPM-TZP-SXT-MEM	*bla*_NDM_	(38) A/C, FIIK, FII	1412
350-W	3 mths	F	Italy	HAC	NSU	9/2/19	*E. coli*	AMK-AMC-FEP-CTX-CAZ-CIP-GEN-IPM-TZP-SXT-MEM	*bla*_NDM_	(39) N, A/C, FII	69
142	2 mths	F	Burundi	HAC	NSU	10/2/17	*E. cloacae*	AMC-FEP-CTX-CAZ-GEN-IPM-TZP-MEM	*bla*_KPC_ + *bla*_NDM_	(40) X3, N	
165	1 mth	F	Italy	HAC	NSU	10/16/17	*E. cloacae*	AMC-FEP-CTX-CAZ-IPM-TZP-MEM	*bla*_NDM_	(41) FII, T, X3, FIA, FIB	
179	5 mths	F	Libya	HAC	NSU	11/20/17	*C. koseri*	AMC-FEP-CTX-CAZ-IPM-TZP-MEM	*bla*_NDM_	(42) FIB, X3	
208-K	1 year	M	Russia	HAC	NSU	2/11/18	*K. oxytoca*	AMK-AMC-CTX-CAZ-GEN-IPM-TZP-MEM	*bla*_NDM_	(43) A/C, FIIK, FIB KN	
234	15 days	M	Italy	HAC	NSU	4/3/18	*K. oxytoca*	AMK-AMC-FEP-CTX-CAZ-GEN-IPM-TZP-MEM	*bla*_KPC_ + *bla*_NDM_	(44) A/C, U	
241	4 mths					4/16/18			*bla*_NDM_		
242	2 mths					4/16/18					
243	1 mth					4/16/18					
248	19 days					4/30/18					
374	1 mth	F	Italy	HAC	NSU	11/11/19	*C. freundii*	AMC-FEP-CTX-CAZ-GEN-IPM-TZP-MEM	*bla*_VIM_	(45) FII, FIB KN, M, HI2	
370	1 mth	M	Italy	HAC	NSU	11/4/19	*C. freundii*	AMC-FEP-CTX-CAZ-IPM-TZP-MEM	*bla*_VIM_	(46) FIB KN, FII, HI2	
375	2 mths	F				11/11/19					
388	3 mths	M				12/10/19					
377	18 days	F	Italy	CAC	NIT	11/12/19	*S. marcescens*	AMK-AMC-FEP-CTX-IPM-TZP-MEM	*bla*_OXA-48_	(47) L	
381	1 mth	M	Italy	HAC	NSU	11/18/19	*C. freundii*	AMK-AMC-FEP-CTX-CAZ-GEN-IPM-TZP-MEM	*bla*_NDM_	(48) A/C, FIB KN, HIB-M, FII, FIB-M	
337	1 year	M	Italy	HAC	NIT	7/15/19	*E. cloacae*	AMC-FEP-CTX-CAZ-CIP-GEN-IPM-TZP-SXT-MEM	*bla*_VIM_	(49) HI2	
389	28 days				NSU	12/16/19					
373	2 days	M	Italy	CAC	SNU	11/7/19	*E. cloacae*	AMC-FEP-CTX-CAZ-GEN-IPM-TZP-SXT-MEM	*bla*_VIM_	UT	
4	8 mths	F	Italy	HAC	NIT	7/25/16	*E. cloacae*	AMC-FEP-CTX-CAZ-GEN-IPM-TZP-MEM	*bla*_VIM_	UT	

^a^ Strains isolated from the same patient were indicated with the same letter (e.g., 137-F and 153-F); ^b^ mths (months); ^c^ CAR (Central African Republic), S. Leone (Sierra Leone); ^d^ HAC (hospital-acquired colonization), CAC (community-acquired colonization); ^e^ NIT (neonatal intensive unit), NSU (neonatal surgery unit), SNU (sub-intensive neonatal unit), CICU (cardiac intensive care unit); ^f^ amikacin (AMK), amoxicillin–clavulanic acid (AMC), cefepime (FEP), cefotaxime (CTX), ceftazidime (CAZ), ciprofloxacin (CIP), gentamicin (GEN), imipenem (IPM), meropenem (MEM), piperacillin–tazobactam (TZP), tigecycline (TGC), trimethoprim-sulfamethoxazole (SXT); ^g^ PBRT (profiles were sorted numerically from 1 to 49); UT = untypeable.

## Data Availability

All data are described within the text. The reads in FASTQ format of ST10555 were uploaded and submitted to the EnteroBase database (https://enterobase.warwick.ac.uk/species/index/ecoli (accessed on 21 February 2022)) and deposited in the Sequence Read Archive (SRA) (GeneBank accession number SUB10115582).
